# Alternation of Remedies

**Published:** 1882-01

**Authors:** 


### HOMŒOPATHIC PHYSICIAN.

A MONTHLY JOURNAL OF MEDICAL SCIENCE.

"If our school ever gives up the strict inductive method of Hahnemann, we are lost, and deserve to be mentioned only as a caricature, in the history of medicine."—CONSTANTINE HERING.

Vol. 2. JANUARY, 1882. No. 1.

### EDITORIAL.

ALTERNATION OF REMEDIES: In discussing the alternation of remedies, we cannot hope, at this late day, to bring forward much that is new—for the practice has, time and again, been shown to be unscientific and unhomœopathic. We feel we cannot open our discussion in a better way than by quoting so distinguished an authority as the late Dr. Dunham. In combatting the views of Dr. J. R. Coxe, Dr. Dunham writes:* "In admitting that the alternation of remedies is incompatible with the theory of a true homœopathic prescription, our colleague has granted all that the opponents of alternation could reasonably ask. * * * But to urge that *because* our practice lags thus behind our theory, we are to rest satisfied with it, nay, to *defend* it and to *conform our theory to it,* is a position that would be fatal to all progress, and which we cannot think of conceding. Nor is the appeal to *experience,* in the sense in which our colleague uses it, valid. He 'has alternated remedies in a practice of twenty-five years, and his practice has been generally successful.' We do not doubt this. But his respected father practiced allopathy for a much longer period, and was a successful practitioner; would *this fact* be a valid argument in favor of *allopathy?*"

We desire to call attention to three points made in this quotation:

1. It is acknowledged that alternation is incompatible with the theory of a true homœopathic prescription. Yet to defend the unsound practice it is alleged that:

---

* See, "Homœopathy, The Science of Therapeutics," p. 156.

2. Alternation is necessary because our practice lags behind our theory. And,

3. Experience proves its usefulness.

(1.) To show that alternation is indeed incompatible with *true Homœopathy*, we need only make a few quotations from the great discoverer and best expounder of our law. On this subject Hahnemann speaks, in his "Organon," so plainly and so forcibly that one cannot mistake his meaning. (Italics ours.)

§ 257. The physician "should never lose sight of this great truth, that of all known remedies there is but *one* that merits a preference before all others, viz.: that whose symptoms bear closest resemblance to the totality of those which characterize the malady."

"§ 272. In the treatment of disease, only *one simple* medicinal substance should be used at a time."

"§ 273. It is impossible to conceive why there should be the least doubt as to whether it is more natural and rational to prescribe a *single* well-known medicine at a time for a disease, or to give a mixture composed of several different medicines."

"§ 274. Perfectly simple, unmixed, and single remedies afford the physician all the advantages he could possibly desire. He is enabled to cure natural diseases safely and permanently through the homœopathic affinity of these artificial morbific potencies; and in obedience to the wise maxim that 'it is useless to apply a multiplicity of means, where simplicity will accomplish the end,' he will never think of giving more than *one simple* medicine at a time. Even taking it for granted that all simple medicines were completely proved with regard to their pure and peculiar action upon the human body, the physician would abstain from mixing and compounding drugs, aware that it is impossible to foresee the variety of effects, that two or more medicines, contained in a mixture, might have; or how the one might modify and counteract the effect of the other, when introduced into the human body. It is equally certain, on the other hand, that a simple medicine, well selected, will by itself be quite sufficient to give relief in diseases whereof the totality of the symptoms is accurately known."

In a foot-note to paragraph 272, already quoted, Hahnemann adds:

"Some homœopathic physicians have tried the plan of administering two medicines at a time, or nearly so, in cases where one of the remedies, seemed to be homœopathic to one portion of the symptoms of the disease, and where a second remedy appeared adapted to the other portion; but I must seriously warn my readers against such an attempt, which will never be necessary even if it should seem proper."

Such were the distinct and forcible utterances of Hahnemann. No comment upon them can be necessary. Yet some advocates of alternation have endeavored to prove that Hahnemann taught alternation as a proper and necessary practice; the quotations just given prove the contrary. To these, we add the testimony of two homœopathists, who have carefully studied Hahnemann's work in the original German.

After a careful study of Hahnemann's words, Dr. Dunham writes: "Indeed, in *theory* and in *practice*, Dr. Bœnninghausen is as decidedly opposed to alternation as we have shown Hahnemann was." Dr. Dunham, moreover shows that in all the cases where Hahnemann seems to advise an alternation of remedies, *a change or alternation of symptoms* is understood. Thus it has been claimed that Hahnemann advised the alternation of Aconite and Coffea; whereas he really says they should be given "as the one or the other is indicated?" How indicated,—by the symptoms?

Another gentleman, Dr. A. Korndœrfer* who has carefully studied the subject, says he wishes "to offer what appears to be such incontrovertible proof of the fact of Hahnemann's continual and consistent opposition to alternation, taken from his own words as found in his works in the original, as would warrant the hope that those who conscientiously differ from us in regard to the use of the single remedy, will not in the future so falsely link his name to that which he so frequently declared to be unscientific, incorrect and needless."

After an elaborate and forcible exposition of Hahnemann's views on alternation, Dr. Korndœrfer concludes as follows:

"Finally, we would remark, that, having carefully reviewed all the quotations which we could obtain from articles written by different alternaters, as published in our journals, the only conclusion to be arrived at seems to be, that either the translations were a perversion of the original, or the quotations have been culled to suit the purpose of the alternater. In fact we feel warranted in asserting that the passages referred to have mostly been garbled so as to suit the pnrpose to which they have been put, whether by the translator, or by the blind follower of an imcompetent interpreter of the works of Hahnemann." In another place, Dr. K. says, "The *best* of books has been wilfully misconstrued; inspired words have been misinterpreted: yea, against their only evident meaning, perverted."

Thus we learn how some disputants argue; how some *homœopaths* prove their practice to be homœopathic.

---

* See *Hahn. Monthly*, vol. 9, p. 320, et seq.

(2.) It is claimed that alternation is necessary, because our practice lags behind our theory. Which is equivalent to admitting that our law is true and effective, but that our skill in applying it is crude, and our knowledge of therapeutics too limited. This being acknowledged, it becomes self-evident that one's skill in practice can never increase except from the study and exercise of it. Could our practice even equal our theory if we deserted it, and so ceased to develope it? Medicine was practiced in that happy-go-lucky way a thousand years ago, but it never made headway till Hahnemann taught that law, not guess, that should guide the physician. But this plea is a mere excuse for idleness or incompetency. If one alternates Aconite and Belladonna to-day, because he cannot say which is *the* simillimum to the case, he should be ashamed to confess this same ignorance to-morrow. If one profited by this practical exposure of his ignorance, as it occurred day by day, he could not long cry—*alternation is necessary.* No man is excusable for making the same mistake *twice.*

All admit that two or more remedies are given because one cannot be found which covers the case completely. This being admitted, the question then naturally arises, cannot the *one* remedy —*i. e., the simillimum*—be found which covers the case completely? Remembering that the remedy must needs to be *most similar* only to the *characteristic* and *peculiar* symptoms of the case, we affirm that this remedy can be found. Two causes of difficulty in finding this *one* remedy suggest themselves : (a) Careless and imperfect examination of the case to be treated; in this way data, totally insufficient for correct prescribing, are procured. (b) An insufficient knowledge of the materia medica, and of Hahnemann's rules for applying it. In giving these two reasons for failure to find the simillimum, we but reiterate Hahnemann's words: * "It is equally certain, on the other hand, that a simple medicine, *well selected*, will, by itself, be quite sufficient to give relief in diseases whereof the *totality* of *the symptoms is accurately known.*" It will be noticed that the two reasons just given prove that if one's practice lags behind the "theory" which our law furnishes, the failure is chargeable to the individual, not to the system. From a careful study of the cases, which are from time to time reported, one must be convinced that the first, of the two reasons here given, is the most com-

* The Organon, 5th edition, sec. 274. Italics ours.

mon cause of failure; much more so than an insufficient knowledge of the materia medica.

(3.) The third argument in favor of alternation was: "Experience proves its usefulness." To deny that cases, even difficult cases, do recover under this treatment, would be unfair. Even Dr. Dunham admits "it is successful, no doubt, at times." But these recoveries are much fewer, slower, and less perfect than under the simillimum remedy. The following case given by Dr. Dunham, illustrates our position clearly and truly:

> "A patient, not long ago, while under a friend's treatment, came under my observation. Her symptoms corresponded exceedingly well with those of *Conium maculatum*. It was a chronic disease of long standing. She had a troublesome constipation, which was sometimes so bad, that it seemed to completely neutralize the good effect which *Conium* was evidently producing. A dose or two of *Opium* 30, would relieve the constipation, and the patient would seem for a while to improve again under *Conium*. This might be called an illustration of what Dr. Drysdale refers to, as the necessity for alternated or intermediate remedies in either 'complications of chronic disease,' or 'exhausted susceptibility.' It was not, however, satisfactory to my friend, nor to myself. He could not regard the regularly recurring constipation as a foreign complication. Believing in the unity of disease, he looked upon it as an integral portion of that patient's sickness, and did not rest contented until he had found a *single remedy* which covered both the symptoms to which *Conium* corresponded and the constipation besides. This remedy was *Alumina*, under which the bowels became, and they have remained, perfectly regular. The patient's improvement, in other respects, was all that could be desired. In this case, as in most cases narrated of cures by alternation, the *Opium* and *Conium*, in alternation, seemed to work very well, and promised to effect a cure in the fullness of time. I doubt not, that if, to all the other histories of cures by alternation, a *sequel* could be written, it would be found that each of these cases has in our materia medica (actual or future), its own particular *Alumina*, which would effect a cure in as few *days* as the most sanguine alternater would expect to accomplish it in *months*."

"The great law, SIMILIA SIMILIBUS CURANTUR, teaches us to select a remedy, the characteristic pathogenetic symptoms of which are very similar to those of the patient. This is a grand generalization, supported by a multitude of facts. We accept it. It takes no heed of *names* of diseases, nor of pathological theories of the seat and origin of diseases. * * * It requires that the symptoms shall be collected and compared with the materia medica *every time a prescription is made*, and that the drug that has produced symptoms most similar to those of the patient shall be chosen and given. This is a true homœopathic prescription." So wrote Carroll Dunham. *We accept this great law, let us then abide by it.*

